# Supplementary material for: Combined Usage of MDK Inhibitor Augments Interferon-γ Anti-Tumor Activity in the SKOV3 Human Ovarian Cancer Cell Line
Source: Biomedicines. 2022 Dec 21;11(1):8. doi: 10.3390/biomedicines11010008 (PMC9855738; doi:10.3390/biomedicines11010008)
Supplement: Supplementary file 1 [file biomedicines-11-00008-s001.zip › biomedicines-2052024-supplementary.pdf]

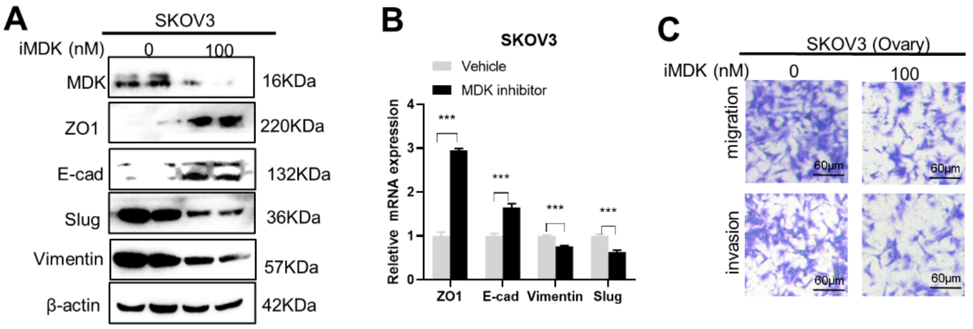

Figure S1. MDK inhibition deactivates the EMT program in SKOV3 cells. A. Western blotting assays to examine the effect of MDK inhibitor (iMDK) on MDK expression and EMT markers in SKOV3 cells. **B.** Real-time qPCR assays to evaluate the effect of MDK inhibition on the expression of EMT markers in SKOV3 cells. **C.** Transwell assays to evaluate the effect of MDK inhibition on migration and invasion properties of SKOV3 cells. Data are represented as mean ± SD, n = 3. ***$p < 0.001$.
